# Development and evaluation of an international teleproctoring program for bronchoscopic procedures

**DOI:** 10.1016/j.isci.2024.111653

**Published:** 2024-12-20

**Authors:** Francesca M. Conway, Anand Tana, Cielito Caneja, James Tonkin, Laura Avanzi, Justin Garner, Christopher M. Orton, Karthi Srikanthan, Ley Chan, John Thornton, Rainer Joachim, Virgile Donet, Philip J. Johnson, Pallav L. Shah

**Affiliations:** 1 National Heart and Lung Institute, Imperial College London, London SW3 6LY, UK; 2 Department of Respiratory Medicine, Chelsea and Westminster NHS Foundation Trust, London SW10 9NH, UK; 3 Department of Respiratory Medicine, Royal Brompton and Harefield Hospital, part of Guys and St Thomas’ NHS Foundation Trust, London, UK; 4 Department of Respiratory Medicine, University Hospitals Bristol and Weston NHS Foundation Trust, Bristol, UK; 5 Nuvaira, Inc, Suite 105 3750 Annapolis Lane North, Minneapolis, MN, USA

**Keywords:** Surgery, Respiratory medicine, Surgical procedure

## Abstract

Medical procedures often require proctoring that is traditionally done face-to-face, but this poses a significant carbon footprint and logistical challenges. Little is known about the safety and efficacy of remote “tele”proctoring, particularly in the respiratory field. The aim was to develop, implement, and evaluate a teleproctoring program for bronchoscopy procedures. Seventy-eight targeted lung denervation procedures were performed at UK hospitals; 20 with teleproctoring, with real-time multimodality data streaming to an off-site proctor; and 58 with on-site proctoring. Procedure completion, procedure duration (bronchoscopy, anesthetic, and fluoroscopy), and safety outcomes (adverse events and device deficiencies) were compared between groups. Teleproctoring offered a safe alternative to on-site proctoring, with little difference in completion rates, procedure durations or safety outcomes. Teleproctoring could be a cost-effective, sustainable and efficient alternative to face-to-face proctoring in appropriate centers. Further work could expand this system to allow remote expert advice to facilitate procedures in clinical practice, and for procedural teaching.

## Highlights

• We designed and evaluated remote “tele”proctoring for bronchoscopy procedures
• We compared tele-proctored procedure outcomes to face-to-face proctoring outcomes
• Procedure completion rate, duration and safety outcomes were similar in both groups
• Teleproctoring in bronchoscopy offered a safe alternative to on-site proctoring

## Graphical abstract

**Figure:**
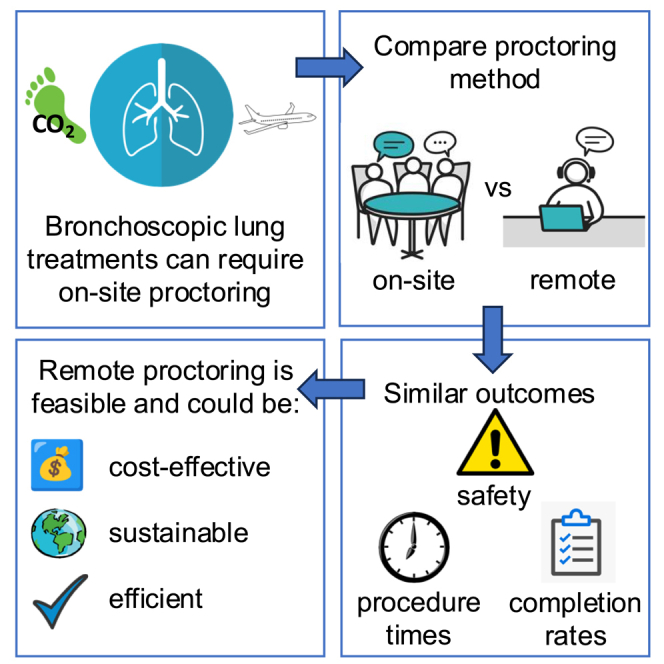

## Introduction

Procedures, particularly those being performed in the context of clinical trials, often require proctoring that is traditionally done on-site face-to-face by a member/members of the company responsible for the equipment or the trial study sponsor. With international restrictions on travel during the COVID-19 pandemic, and the significant carbon footprint of clinical trials, it is relevant and timely to consider whether procedures that were traditionally proctored on site could be proctored remotely to reduce the need for travel. Patients were enrolled into a clinical trial of a bronchoscopic therapy for chronic obstructive pulmonary disease (COPD) that would usually be proctored by the study sponsor on site. We developed, implemented and evaluated an international teleproctoring program for this bronchoscopic treatment. While this work is relevant to a bronchoscopic procedure, the set up and technology would likely also be relevant to other procedures, and if effective, the set-up could potentially be expanded beyond just the proctoring field to support clinical care of patients where real time expert advice is not readily available at the center performing the procedure. To our knowledge, this is the first pilot program to be employed in this procedural field and one of the largest teleproctoring pilots across any specialty.

Targeted lung denervation (TLD) is a bronchoscopic procedure being investigated in clinical trials for airways disease. The AIRFLOW-3 trial (NCT03639051) is a multinational, phase 3 randomized controlled trial investigating the effect of TLD on exacerbations in COPD and the study design has been previously described.^1^ The treatment involves delivery of radiofrequency energy bronchoscopically to the airways to disrupt the parasympathetic nerve supply, which is dysregulated in COPD. As such, it is an ideal example of a bronchoscopic procedure being investigated, which requires proctoring by industry and in which to evaluate the safety and feasibility of teleproctoring.

**Figure 1:**
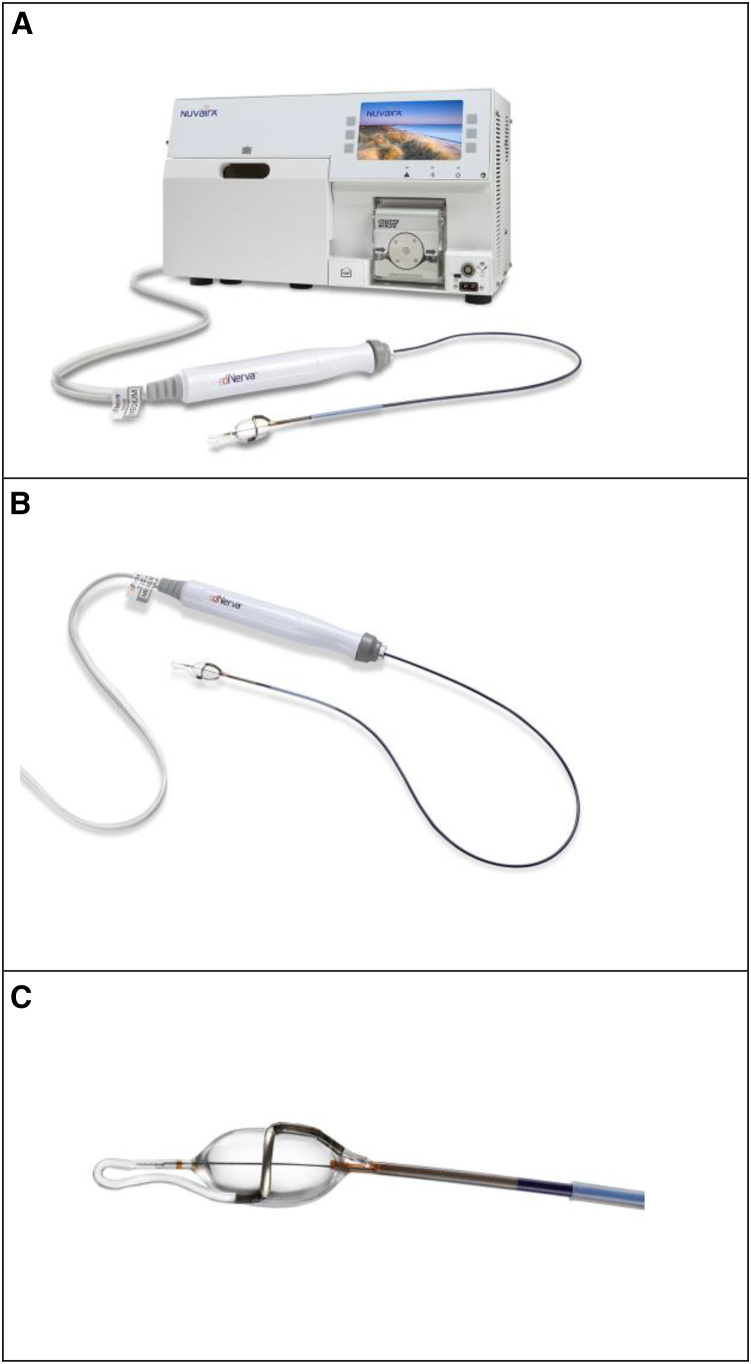
Images of equipment for TLD procedure (A) TLD console connected to catheter. (B) TLD catheter. (C) Close up of the balloon and electrode on the TLD catheter.

Briefly, the bronchoscopy procedure is performed under general anesthetic in a bronchoscopy suite or theater in select centers, our centers being in the UK. The TLD equipment consists of a disposable catheter and a console with a user interface, which allows for energy selection and delivery. The disposable catheter consists of an umbilical that connects to the console at one end, and a balloon and electrode that applies the treatment in the airway at the opposite end. A cooling plate within the console cools dextrose which is then circulated through the catheter inflating the balloon at the end. The console pump expels surplus air ensuring that the balloon is filled with dextrose and is turgid. Images of the console and catheter are shown in Figure 1.

### Research in context

#### Evidence before this study

**Figure 2:**
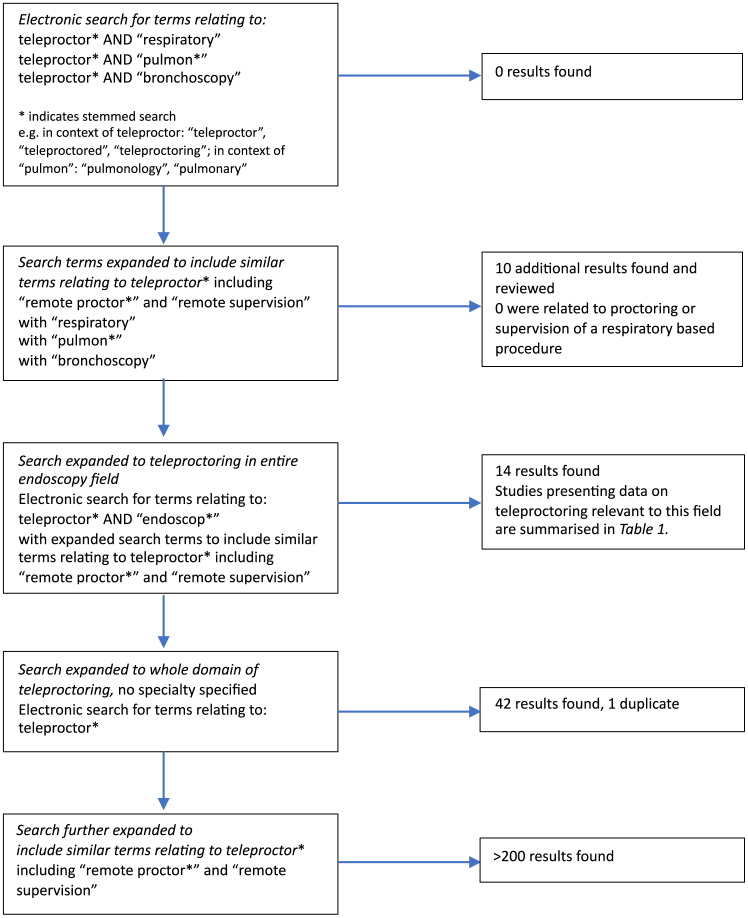
Search strategy for literature review

Teleproctoring has been explored in some procedural fields previously with case reports and case series presented.^2^ Most work to date has been in small numbers and to our knowledge there has not been any work evaluating teleproctoring in bronchoscopy procedures or the respiratory field as a whole. A systematic search of PubMed electronic database for studies that included teleproctoring of respiratory procedures published without date or language restriction was conducted between February 2024 and April 2024, additional searches were performed in August 2024. A schematic displaying the search criteria is displayed in Figure 2. Keyword and stemmed search terms relating to teleproctor (“teleproctor,” “teleproctored,” and “teleproctoring”) with “respiratory” OR “pulmonology” OR “bronchoscopy” revealed no results. The search terms were expanded to include other related terms such as “remote proctor” and “remote supervision” that found 10 results but on review of them they were not relevant to teleproctoring of respiratory procedures. Search terms relating to teleproctor (as aforementioned) with “endoscopy” yielded 14 results. The original research articles related to the fields of gastrointestinal (GI) procedures, simulation-based laparoscopic training, and sinus surgery and a summary of those relevant to this work is provided in Table 1.

**Table 1:** Results of literature review to identify previous work in teleproctoring in endoscopy

Authors	Title	Aim	Method	Results
Torabi J, Abeshouse M et al.^3^	Remote training and teleproctoring in gastrointestinal endoscopy for practicing surgeon in rural Uganda.	Create “a remotely proctored endoscopy training program for a surgeon practicing in an area devoid of endoscopic capabilities”	Retrospective case series between February 2020 and December 2022 in Uganda of a surgeon performing endoscopy cases	The surgeon underwent remote proctoring for endoscopy procedures (*n* = 139 cases), before independently performing endoscopy, followed by performing therapeutic endoscopy (*n* = 43) under remote guidance. All procedures were completed without complications.
Asfaw ZK, Todd R et al.^4^	Use of virtual platform for delivery of simulation-based laparoscopic training curriculum in LMICs.	To “explore the feasibility and efficacy of virtual laparoscopic simulation training in resource-limited settings”	A simulation-based laparoscopic training curriculum for surgeons was adapted to a remote format. 36 participants were enrolled in Mexico and Costa Rica. Surgeons’ performance was assessed.	“Respondents reported that the program was a good use of their time and that education via telesimulation was easily reproduced.” After the course, residents completed all tasks of the exam quicker relative to their performance prior.
Galvao Neto M, Jerez J et al.^5^	Learning process effectiveness during the COVID-19 pandemic: Teleproctoring advanced endoscopic skills by training endoscopists in endoscopic sleeve gastroplasty procedure.	To evaluate a remote training program for endoscopic sleeve gastroplasty	“Ten patients underwent an endoscopic sleeve gastroplasty procedure guided by a proctor expert using an online platform”	“All cases were safely performed with no serious adverse events under teleproctoring.” The average surgical and suturing times significantly decreased during the training model. Conclusion: “the proposed teleproctoring program was effective in delivering advanced endoscopic skills.”
Burgess LP, Syms MJ et al.^6^	Telemedicine: teleproctored endoscopic sinus surgery.	To evaluate the safety and feasibility of teleproctored surgery as compared with the current standard of care for endoscopic sinus surgery	Evaluated a residency training program comparing 42 patients having conventionally proctored endoscopic sinus surgery cases with 45 patients have teleproctored surgery where the supervising surgeon supervised through audiovisual teleconferencing (VTC) in a control room.	“3 teleproctored cases required faculty intervention: 2 for surgical difficulty, one for VTC problems. Teleproctored cases took 3.87 minutes longer per side (28.54 vs. 24.67 min, *p* <0.024), a 16% increase. This was thought to be a result of nuances of VTC proctoring.”
Rafiq A, Moore JA et al.^7^	Asynchronous confirmation of anatomical landmarks by optical capture in open surgery.	To establish a “validation model … to assess the capabilities of current technologies to conduct effective instruction of surgical procedures in a remote location relative to the actual surgical procedure”	“23 unilateral thyroid dissections in 13 patients using a laparoscope affixed to a stationary robotic arm were videotaped.” Surgical trainees were required to confirm anatomical landmarks	“The anatomical structures were clearly identifiable in an instructional environment”
Okrainec A, Vassiliou M et al.^8^	Feasibility of remote administration of the Fundamentals of Laparoscopic Surgery (FLS) skills test.	To “assess the feasibility of remotely administering and scoring the Fundamentals of Laparoscopic Surgery (FLS) examination using live videoconferencing compared with standard onsite testing”	“An official FLS proctor administered and scored the FLS exam remotely while another onsite proctor provided a live score of participants' performance”	“The scores of the remote and onsite proctors showed excellent interrater reliability in the total FLS”
Okrainec A, Vassiliou M et al.^9^	Remote FLS testing in the real world: ready for “prime time.”	To “validate the integrity and validity of the FLS manual skills examination administered remotely in a real-world environment according to FLS testing protocols”	20 participants completed the test. Participants entered a testing room connected via Skype to a separate room with a proctor who administered and scored the test remotely, while an on-site proctor was present in the room as a control.	There was no significant difference between scores by the remote proctor compared to the on-site proctor. “One critical error was missed by the remote proctor, but this would not have affected the test outcome”. Participants reported being highly satisfied.
Hiatt JR, Shabot MM et al.^10^	Acceptability of compressed video for remote surgical proctoring.	“To determine the clinical acceptability of various levels of video compression for remote proctoring of laparoscopic surgical procedures”	Observational, controlled study. Surgical videos underwent various levels of data compression for digital transmission and display and were shown to observers who commented on acceptability of the videos after compression.	“With proper video compression, remote proctoring of laparoscopic procedures may be performed with standard 1.5-megabit/second telecommunication data lines and services.”
Kambakamba P, Naiem A et al.^11^	Applying augmented reality in teaching of surgical residents-telementoring, a “stress-free” way to surgical autonomy?	To “analyze the feasibility of augmented reality (AR) application in surgical training and to assess its impact on intraoperative stress”	5 doctors performed various surgical procedures semi-autonomously versus with full remote supervision via augmented reality (AR) glasses, worn by the resident in theater.	AR remote assistance “showed satisfactory applicability”. There were lower markers of stress in the AR participants compared to in the semi-autonomous group
Kim CY, Etemad B et al.^12^	Remote clinical assessment of gastrointestinal endoscopy (tele-endoscopy): an initial experience.	To evaluate the quality of tele-endoscopy for cancer screening.	10 patients undergoing endoscopic procedures were observed by the endoscopist and a remote observer. “Findings by both were compared for concordance on malignant or premalignant lesions.”	“The image quality was adequate to support remote diagnosis of GI cancer and abnormal lesions by an experienced observer” … “Tele-endoscopy is both adequate and feasible for diagnosis of most gastrointestinal lesions. Subtle lesions still may be missed in our current setup.”

We thereafter conducted a further search of teleproctoring without specifying a field it was employed in, using just the keyword and stemmed searches as relevant relating to teleproctor, for articles published with no date or language restrictions, which yielded 42 results, of which 1 was a duplicate. Expansion of the search criteria to include “remote supervision” and “remote proctor” yielded more than 200 results, with a variety of evaluations performed. A significant portion of these were review articles. Of the original research articles, some evaluated the role of teleproctoring and remote medicine in an educational capacity for teaching, or remote supervision and monitoring of patients outside of a procedural setting. Of the original articles using teleproctoring for procedures, some were simulation-based and some were real life. Few studies directly compared teleproctoring with face to face proctoring of live cases, which we consider to be the most applicable and relevant to real life procedures, and with the exception of a few, a high number of those that did were small case series where teleproctoring was employed for 10 or fewer cases.

One group evaluated the use of teleproctoring in implantation of a new flow diverter in three patients undergoing treatment for intracranial aneurysms in the context of a clinical trial.^13^ The authors report that all devices were implanted as desired with no complications and that the procedure durations and radiologic dose was similar to routine cases done in their center.^13^ A further group evaluated outcomes of teleproctoring for robotic gynecologic surgery; 7 cases were completed with teleproctoring and compared to 59 historical cases that had been proctored in person.^14^ Surgeons reported high satisfaction with teleproctoring and there were no major technological issues. Tele-proctored cases were shorter than controls, with no difference in complication rates. A further group presents a case where teleproctoring enabled successful performance of a new structural heart interventional procedure.^15^ A group in the US evaluated the role of remote technology within the bronchoscopy field from an educational perspective, evaluating telementoring and remote learning in bronchoscopy education.^16^

The catheter with electrode is introduced through the working channel of the bronchoscope and into the patient’s airways by the bronchoscopist under direct visualization with white light bronchoscopy. The bronchoscopist navigates to the target treatment zones which are the right and left main bronchus, the balloon is inflated (filled with dextrose), the position is confirmed fluoroscopically and the distance between the electrode and the balloon containing radio-opaque contrast that has been placed within the esophagus is measured. Once a satisfactory position is obtained, the bronchoscopist delivers radiofrequency energy or “sham energy” as per trial randomization via the console to the patient’s airways circumferentially in 4 quadrants bilaterally. It is imperative to get perfect balloon contact with the airway wall to ensure the airway mucosa is cooled during the energy application to protect the airway epithelium. Fluoroscopy confirms the correct position of the electrode prior to every activation of energy. The procedure is completed by an airway inspection. The AIRFLOW-3 study is sponsored and proctored by Nuvaira who own the lung denervation system.

The COVID-19 pandemic restricted travel for proctors and halted or delayed clinical trials, and the introduction of new medical devices. Nuvaira, the study sponsor of the AIRFLOW-3 clinical trial, is based largely in the USA with some staff throughout Europe, but no staff based in the UK. Travel restrictions during this period varied from proctors not being able to enter the UK, having to isolate, or not being allowed to enter clinical areas of the hospital, thus meaning that TLD procedures had to be temporarily halted.

Patients with COPD who were enrolled in the study and eligible were ready to undergo their procedures, and there are often narrow windows of opportunities to perform the bronchoscopic treatment due to clinical considerations. It was therefore important that procedures were performed promptly at the safest time to do so without logistical delays, particularly considering that initial work has suggested that TLD may reduce the rate of lung function decline^17^ and exacerbation frequency.^18^^,^^19^ If this effect is confirmed, the cost to the patient of missing out on a potentially helpful procedure is therefore significant. Extension of trial timelines also results in delays to device evaluation, regulatory approvals and the product getting to market. There are also business-related and financial implications of prolonging a large multinational study. Together, these factors were motivators to resume treatments promptly and safely in the context of the COVID-19 pandemic and travel restrictions. The team elected to develop and implement a teleproctoring program to allow the ongoing smooth running of procedures, thus reducing delays to patients and the clinical trial as a whole. Another driving factor for the implementation of teleproctoring programs stems from the need to reduce the significant carbon footprint incurred by clinical trials. A study in 2009 of 12 pragmatic randomized controlled trials showed that the average CO_2_ emission generated by the trials was 78.4 tonnes, which is equivalent to that produced by 9 people in one year in the UK.^20^ Air travel is a significant contributor to this, with one study showing that 23% of the greenhouse emissions generated from the CRASH trial (a multicentre international trial of the effect of corticosteroids in adults with head injury)^21^ were related to trial related travel.^22^ Remote proctoring offers an opportunity to reduce travel associated with clinical trials, and with many study sponsors having targets to become carbon neutral, this is an avenue worth pursuing.

## Results

Seventy-eight TLD procedures performed by the interventional bronchoscopy team in the UK as part of the AIRFLOW-3 trial are included in this work. Twenty were done with teleproctoring and the remaining 58 were proctored using traditional on-site face-to-face proctoring. The breakdown of procedure types is displayed in Table 2. There were no significant differences in baseline characteristics between the face-to-face group and the teleproctoring group (Table 3). For patients undergoing cross over procedures, baseline demographics from the initial screening visit were used.

**Table 2:** Break down of procedure types by group

	Face to face group *N* = 58	Teleproctor group *N* = 20
Overall number of procedures by study arm	Sham = 21 (36%) Treatment = 37 (64%)	Sham = 9 (45%) Treatment = 11 (55%)
Roll in procedures	3	0
Randomization procedures	Total = 43 Sham = 21 (49% of randomized) Treatment = 22 (51% of randomized)	Total = 17 Sham = 9 (53% of randomized) Treatment = 8 (47% of randomized)
Cross over procedures	12	3

*N* = number of patients.

**Table 3:** Patient baseline characteristics

	Face to face proctor group *N* = 58	Teleproctor group *N* = 20	Difference between groups
**Demographic characteristic**			

Age (years)	68.3 ± 7.16	67.5 ± 5.82	*p* value = 0.42 (95% CI: −2.0 to 5.0)
Male sex n (%)	23/58 (39.7%)	10/20 (50%)	*p* value = 0.59
Body mass index (BMI)	26.5 ± 4.60	28.6 ± 3.85	*p* value = 0.077 (95% CI: −4.0 to 0.00)
Smoking history (pack years)	44.2 ± 21.6	45.5 ± 28.1	*p* value = 0.66 (95% CI: −10 to 13.0)

**Lung function**			

FEV_1_ value (L)	1.01 ± 0.37	1.00 ± 0.29	*p* value = 0.76 (95% CI: −0.17 to 0.16)
FEV_1_ percent of predicted value	40.7% ± 12.1%	38.3% ± 7.9%	*p* value = 0.85 (95% CI: −4.0 to 6.0)
FVC value (L)	3.10 ± 1.00	3.06 ± 0.89	*p* value = 0.89 (95% CI: −0.52 to 0.50)
FVC percent of predicted value	94.6% ± 17.8%	89.0% ± 13.0%	*p* value = 0.14 (95% CI: −1.88 to 13.1)
Residual volume (L)	3.85 ± 0.80	3.97% ± 0.84	*p* value = 0.57 (95% CI: −0.56 to 0.31)
Total lung capacity (L)	6.93 ± 1.38	6.94 ± 1.35	*p* value = 0.96 (95% CI: −0.73 to 0.70)

**COPD symptom baseline**			

COPD assessment test (CAT) score	25.2 ± 6.4	24.9 ± 7.3	*p* value = 0.86 (95% CI: −3.43 to 4.07)

Data are presented as mean ± standard deviation.

CI, confidence interval; FEV1, forced expiratory volume in the first one second; FVC, forced vital capacity; *N*, number of patients.

### Set up and development of the remote proctoring program

**Figure 3:**
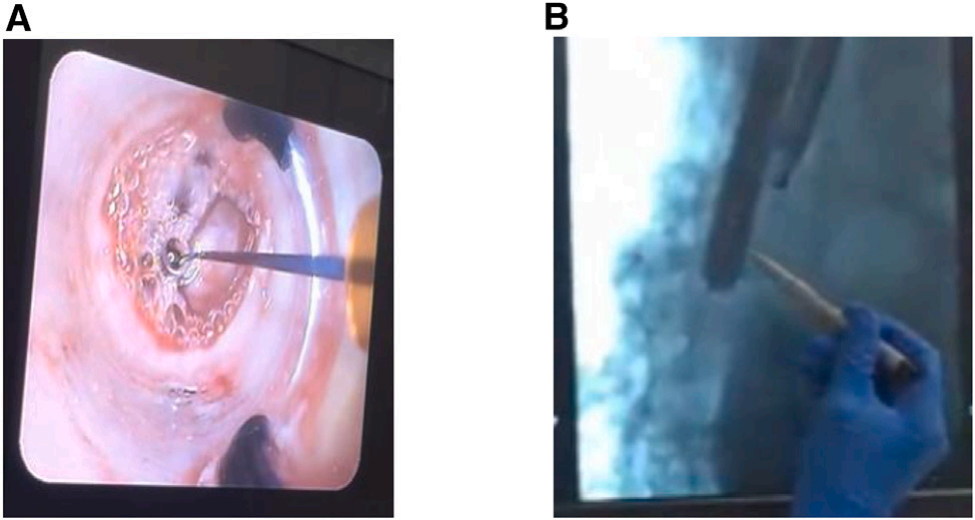
Endobronchial image screenshot from the teleproctor’s screen (A) This is an image of the endobronchial view from the bronchoscope camera as seen on the teleproctor’s screen. The teleproctor has a clear view of the entire bronchoscopy screen, including the patient’s airways, and the balloon and electrode of the catheter. This is the same view that is seen by the on-site treating physician team. Image not to scale. (B) Fluoroscopy image screenshot from the teleproctor’s screen. This is an image of the fluoroscopy view projected within the bronchoscopy suite and to the teleproctor. The X-ray of the patient’s chest is well visualized including the bronchoscope and the contrast-filled balloon within the esophagus. (Image not to scale. Image has been cropped to anonymize it).

Formal training had already been provided by the study sponsor to the hospital bronchoscopy team on performing and assisting in the TLD and sham procedure prior to the development of the teleproctoring program. Discussions were held between the hospital team and the study sponsor to assess what equipment would be needed. Prior to the commencement of the teleproctoring program, a test designed by the study sponsor was undertaken by the hospital team, to ensure competence in all procedural aspects without assistance from an on-site proctor, including clinical aspects such as performance of the procedure, and additionally technical aspects related to equipment set up and trouble shooting. A test run was also performed to check the audiovisual connection, with various equipment trialled to get the optimal results, including different internet connections and different audiovisual devices. Backup solutions were also established in the case of a loss of connectivity to ensure in such an instance, the connectivity could be rapidly restored without impacting the procedure. Initial tested equipment included hospital computer equipment, sharing of screens with information, and portable devices including mobile phones and tablet devices, using handheld devices for specific requirements such as closer views or views requiring motion, and devices mounted on tripods for stationary views. Different audio proposals included use of a speaker through which the proctor could state instructions, 2 way audio communication through an audio or video call audible to the entire room, or headphones directly to one person who acted as a liaison to the rest of the team. The remote proctor had access to visualize the fluoroscopy screen, the position of the bronchoscopist’s hands via a mobile camera, and the endobronchial bronchoscopic view via a mounted camera that could also be moved as required. An example of images as visualized by the teleproctor is provided in Figure 3. The set up of the bronchoscopy suite was further refined throughout the study. Increasingly complex systems including a system being developed at the Royal Brompton Hospital included multiple cameras in the room and a large central screen with multicomponent imaging side by side including the endobronchial view and the fluoroscopy screen where measurements could be directly measured digitally and stored.

The teleproctor was virtually present from the preparation and set up of the equipment through to the end of the procedure, equivalent to their in-person presence, and worked to the same quality and safety standards as if they were on site. The same aspects of the procedure were checked virtually by the teleproctor as were checked in face to face procedures by the proctor; for example, balloon position prior to energy activation, safety fluoroscopy check part way through activation to ensure stability of the balloon during energy delivery, and inspection of the airway after each delivery to evaluate airway epithelial integrity.

All standing study protocol and company standard operating procedures regarding physician training, staff training, and proctoring were met in the context of the remote proctoring plan that was put in place. This included prior tests of intraprocedural communication mediums for proctors/procedure teams, and contingencies for loss of communication. As a result, no additional legal liability specific to the remote proctoring was added to the procedure.

### Procedure successful completion rates

100% of procedures in both the face-to-face proctoring and teleproctoring group were successfully completed, 58/58 and 20/20 procedures respectively (Table 4). Successful completion was defined as the proctor and bronchoscopist being satisfied that all quadrants amenable to denervation treatment were treated with either TLD or sham as per trial randomization.

**Table 4:** Outcomes from face to face and teleproctoring of TLD procedures

	Face to face group *N* = 58	Teleproctor group *N* = 20	Difference between groups
Successful completion	58/58 (100%)	20/20 (100%)	N/A
Mean bronchoscopy duration (minutes) ± SD	70.6 ± 18.4	76.2 ± 17.4	*p* value = 0.23 95% CI: −15.0 to 3.7
Median bronchoscopy duration (minutes) (IQR)	67.5 (57.5–83.75)	77 (60–87.75)	
Mean anesthetic duration (minutes) ± SD	84.1 ± 19.6	88.1 ± 16.8	*p* value = 0.28 95% CI: −15.0 to 5.0
Median anesthetic duration (minutes) (IQR)	80 (72.25–97.5)	85.5 (75–101.25)	
Mean fluoroscopy duration (seconds) (treatment procedures only) ± SD	241 ± 94	228 ± 89	*p* value = 0.81 95% CI: −45.0 to 64.0
Median fluoroscopy duration (seconds) (treatment procedures only) (IQR)	223 (181–275)	224 (177–251.5)	

CI, confidence interval; IQR, interquartile range; SD, standard deviation.

### Procedure times

Bronchoscopy times are presented in Table 4. The mean bronchoscopy duration in the face-to-face group was 70.6 ± 18.4 min, around five and a half minutes shorter than the 76.20 ± 17.4 min in the teleproctored group, which was not a statistically significant difference (*p* = 0.23). The mean anesthetic duration was 4 min shorter in the face-to-face group at 84.1 ± 19.6 min, compared to 88.1 ± 16.8 min in the teleproctored group, which was also not statistically significant (*p* = 0.28). The mean fluoroscopy duration was shorter in the teleproctored group at 228 ± 89 s, compared to 241 ± 94 s in the face-to-face group, which was not statistically significant (*p* = 0.81).

### Device or procedure related adverse events

Events included were those which could have been potentially related to the proctoring method occurring peri-procedure or in the post procedure follow up, up to 3 months after the procedure. Events such as COPD exacerbations were not included as this is an expected event following bronchoscopy with TLD/sham procedure and proctoring method is not considered to be relevant to this. Events are only included if reported as causal, probable, or possible relation to procedure by the bronchoscopy team. Events are displayed in Table 5.

**Table 5:** Adverse event rates where proctoring method could be relevant

	Face to face group *N* = 58 n/N (%)	Teleproctor group *N* = 20 n/N (%)
Airway ulceration	1/58 (1.7%)	0 (0%)
Acute respiratory failure post-procedure	5/58 (8.6%)	1/20 (5%)
Intraprocedural airway hemorrhage	0 (0%)	0 (0%)
Hemoptysis within 3 months of procedure	1/58 (1.7%)	0 (0%)
Pneumothorax within 3 months of procedure	0 (0%)	0 (0%)
Bronchial perforation/broncho-esophageal fistula within 3 months of procedure	0 (0%)	0 (0%)
Acute cardiac event within 3 months of procedure (possible)	1/58 (1.7%)	0 (0%)
Miscellaneous procedure related	1/58 (1.7%) Oral bruising post intubation requiring direct visualization	0 (0%)
Mortality during or within 3 months of procedure	0 (0%)	0 (0%)

### Device deficiencies

Device deficiencies were defined as per international standards set out in the ISO standard (the International Organization for Standardization): ISO 14155:2020: “Clinical investigation of medical devices for human subjects—Good clinical practice” as: “*inadequacy of a medical device with respect to its identity, quality, durability, reliability, usability, safety or performance.*”^23^ We applied this definition to deficiencies of the console, catheter including balloon and electrode, or cooling plate. Device deficiencies occurring during equipment set up or intraprocedurally were recorded. The set up of the system is described previously. 9/58 (15.5%) of face-to-face procedures experienced at least one device deficiency, whereas in the teleproctoring group there were 5/20 (25%) of procedures with at least one (Table 6). These included issues specific to the catheter utilized that included catheters being stiff or difficult to maneuver or issues with balloon inflation. Other issues included technical issues with the console including connection or user interface issues. These are described for each procedure where at least 1 device deficiency occurred in Table 7 with the impact to patient rated (independently by the treating hospital team, and the study sponsor), and the action required to resolve the issue outlined. All device deficiencies were reviewed by the industry study sponsor and by the bronchoscopy team independently and none of these were considered to be affected by the proctoring method and would have likely occurred regardless of this.

**Table 6:** Device deficiencies occurring in both groups

	Face to face group *N* = 58 n/N (%)	Teleproctor group *N* = 20 n/N (%)	Difference between groups
Median number of device deficiencies per procedure in each group	0	0	N/A
Number of procedures where 1 or more device deficiency occurred	9/58 (15.5%)	5/20 (25%)	*p* value = 0.33 95% CI: 0.14 to 2.45
Number of procedures where a device deficiency occurred that was considered to be related to the proctoring method	0/58 (0%)	0/20 (0%)	N/A

**Table 7:** Description of all procedures where a device deficiency occurred

Teleproctor group			
Recorded device deficiency/deficiencies	Impact to Device	Impact to Patient	Action(s) taken to resolve
Catheter malfunction	Balloon inflation	no/low	replacement of catheter(s), re-set up of console/catheter set
Catheter malfunction	Balloon inflation	no/low	replacement of catheter(s)
Catheter malfunction	Catheter positioning	no/low	replacement of catheter(s)
Catheter malfunction	Balloon inflation	no/low	replacement of catheter(s)
Catheter malfunction	Catheter positioning	no/low	replacement of catheter(s)

**Face to face group**			

Catheter malfunction	Catheter positioning	no/low	replacement of catheter(s)
Catheter malfunction	Balloon inflation	no/low	replacement of catheter(s)
Catheter malfunction	Balloon inflation	no/low	replacement of catheter(s)
Catheter malfunction	Balloon inflation	no/low	replacement of catheter(s)
Console malfunction	Console display	no/low	console reboot
Console malfunction	Console display	no/low	deflation and inflation of balloon had to be repeated
Catheter malfunction	Catheter positioning	no/low	deflation and inflation of balloon had to be repeated
Catheter malfunction	Balloon inflation	no/low	replacement of catheter(s), re-set up of console/catheter set and cooling plate
Catheter malfunction	Catheter/balloon	no/low	replacement of catheter(s)

### Teleproctoring system issues

There were no significant technology failures in the teleproctoring group restricting the ability of the proctor and the bronchoscopist to communicate.

## Discussion

Clinical and research bronchoscopic procedures involving new equipment or medical devices are often supported in person by personnel from the industry responsible for the devices and technology and/or the study sponsor. This work has provided useful information on setting up a teleproctoring system in this field and the value it could offer in bronchoscopy procedures. Teleproctoring has the potential to reduce the need for the proctor to be present in the bronchoscopy suite; which was valuable during times of travel restrictions during the COVID-19 pandemic, and also could potentially reduce the carbon footprint of clinical trials. Additionally this could be extended outside of bronchoscopy and even the respiratory field to a variety of different procedures. Furthermore, this approach of using real time audiovisual links between staff in different countries to successfully observe and where required provide assistance could be expanded beyond the field of proctoring. It could be a useful tool for clinicians in remote areas to be able to seek input from experts at other centers in real time to assist during a procedure. If performed safely and within rigorous guidelines, this could facilitate centers performing procedures where this may not otherwise have been possible. It could also be a very valuable approach to procedural training without the need for learners to travel to the center where the teacher(s) are based. The safety of using this method would need to be evaluated in emergency or urgent situations and in centers individually to ensure that the connectivity is adequate; however, we did not encounter any significant technical issues restricting the ability for the proctor to have two-way communication to safely monitor and observe the procedure and directly interact with the bronchoscopist.

This work suggests that remote proctoring offered a safe alternative to on-site proctoring of these interventional bronchoscopy procedures. It was expected that teleproctoring could increase procedure durations, due to the need for additional communication between the bronchoscopist and proctor above what would normally be required and to allow time for the proctor to view the different visual fields. However, it was reassuring to note that teleproctoring did not drastically increase the bronchoscopy or anesthetic duration, as particularly in patients with lung disease, significant prolongation of the procedure would be undesirable. As well as reducing the need for travel, this option could prove to be cost-effective. There were no specific additional costs incurred for this program to be set up, as the audiovisual equipment was already available in the hospital, so considering the travel cost savings, this could be more economical than face-to-face proctoring. The carbon savings have not been directly calculated but the carbon associated with travel was undoubtably reduced.

### Limitations of the study

The strength of this work lies in this being one of the largest studies that we have found to evaluate teleproctoring for procedures, and the first evaluation of teleproctoring in bronchoscopy procedures and within the respiratory field. Compared with most other work in teleproctoring, some of which are single case reports or small case series, we evaluated a relatively high number of patients in this analysis, although we do acknowledge that the number in the teleproctoring arm was lower than in the face-to-face arm. A further advantage was that all bronchoscopy procedures were completed by the same bronchoscopy team using the same equipment thus eliminating the risk of bronchoscopist skill level and experience biasing the procedure durations or complication rates.

It should be noted that the bronchoscopy team were very experienced and had performed more than 90 TLD procedures since March 2016. It would be relevant to test this system also in centers with less experience and more limited skills to see if safety is still maintained. The procedures were also teleproctored by proctors with significant experience in proctoring this procedure. High quality audiovisual equipment allowed the communication to be in real time between the proctor and bronchoscopist. All cases were also reviewed prior to the procedures being performed by the proctoring team including CT thorax imaging and also separately by the bronchoscopy team with the aim to anticipate and identify any potential issues that could arise during the case and mitigate these. This is routine practice for this team prior to performing each case for this procedure regardless of proctoring method.

In summary, remote proctoring offered a safe and effective alternative to on-site proctoring of TLD bronchoscopy procedures. It could prove to be a cost-effective, sustainable and efficient alternative to face to-face proctoring in appropriately selected centers, reducing the carbon footprint of clinical trials and mitigating delays to clinical trials and patients due to travel logistics.

## Resource availability

### Lead contact

Requests for further information and resources should be directed to and will be fulfilled by the lead contact, Francesca M. Conway (francesca.conway07@imperial.ac.uk).

### Materials availability

This study did not generate new unique reagents.

### Data and code availability

• Data reported in this paper will be shared by the lead contact upon request.
• This paper does not report original code.
• Any additional information required to reanalyze the data reported in this paper is available from the lead contact upon request.

## Acknowledgments

The study sponsor, Nuvaira, were responsible for the study design of the AIRFLOW-3 trial and involved in the design and development of the teleproctoring pilot program presented in this work. They were also involved in contributing to the writing of this manuscript and in the decision to submit the paper for publication. The AIRFLOW-3 study of Targeted Lung Denervation is sponsored by Nuvaira, Inc, Minneapolis, USA. F.M.C. has received a 10.13039/501100000265Medical Research Council fellowship award to support her PhD, grant ref.: MR/V00171X/1.

## Author contributions

The teleproctoring program was designed, developed and implemented collaboratively by P.L.S., F.M.C., and C.C. from the Chelsea and Westminster Hospital team and Nuvaira (R.J., V.D., and P.J.J.). F.M.C. performed literature search. Study conception and design was by F.M.C., P.L.S., and P.J.J. J. Thornton provided anesthetic support. Proctoring of procedures was by R.J. and V.D. F.M.C., A.T., C.C., J. Tonkin, L.A., J.G., C.O., L.C., and K.S. were involved in data acquisition. Data analysis, review and interpretation was performed by F.M.C., P.L.S., and P.J.J. The first draft of the manuscript was written by F.M.C. with input from P.L.S. and P.J.J., with review and edit by F.M.C., P.L.S., and P.J.J. All authors approved the final version of the manuscript prior to submission. F.M.C., P.J.J., and P.L.S. have directly accessed and verified the underlying data reported in the manuscript.

## Declaration of interests

Institution: Royal Brompton Hospital and Chelsea and Westminster Hospital have been reimbursed for clinical trial expenses (Airflow 3 Trial).

F.M.C. has received a pre-doctoral fellowship from the medical research council to support her PhD, which is relevant to this work. P.J.J., R.J., and V.D. are paid employees of Nuvaira.

## STAR★Methods

### Experimental model and study participant details

#### Experimental models

Experimental models relating to animal, plant, cell line and microbes are not relevant to this work. The influence of sex on our outcomes was not assessed as the cohort was analyzed as a whole, we did not consider there to be an indication or scientific rationale for subgroup analysis by sex for the purpose of this work.

#### Study participants

Patients enrolled in the AIRFLOW-3 trial had provided written informed consent to participate in the AIRFLOW-3 trial and undergo a bronchoscopic procedure as per trial randomization. The AIRFLOW-3 study received ethics approval from East of England – Cambridge East (REC No. 19/EE/0080) Ethics Committee and Health Research Authority. This trial is registered on ClinicalTrials.gov (NCT03639051). The initial inclusion and exclusion criteria for the AIRFLOW-3 study has been previously described,^1^ although some amendments were made to these as the trial progressed, most notably a widening of the FEV1 inclusion criteria. Patients included in this work were sequentially treated with TLD or sham procedure at the Chelsea and Westminster Hospital (with one face-to-face case being performed at the Royal Brompton Hospital to aid with logistics, with the same hospital physician and support team, and the same TLD equipment), London, UK, between July 2019 and July 2023. This included “roll-in” (three patients who underwent non-randomized treatment procedures), randomized and cross-over procedures. Randomization to the TLD treatment group compared to the sham group was conducted as part of the whole AIRFLOW-3 multinational clinical trial, with equal allocation (1:1) into two arms: TLD therapy plus optimal medical care (Active Treatment) and sham procedure plus optimal medical care (Sham Control). Randomization of subjects was stratified based on investigational site, participation in a pulmonary rehabilitation maintenance program and baseline use of an inhaled corticosteroid at the time of enrollment. Where there was onsite proctoring available, procedures were performed with onsite face to face proctoring. Following set-up of the remote proctoring program, patients whose procedures occurred when it was not possible for the proctor to be present in the bronchoscopy suite in person, had their procedures proctored remotely.

### Method details

#### Aims

The aim of this work was to develop, implement and evaluate the safety and efficacy of a teleproctoring program for bronchoscopic procedures.

#### Methodology

This was a pilot project to implement a teleproctoring program for a bronchoscopy treatment for COPD being evaluated in clinical trials. The interventional bronchoscopy team and Nuvaira, the study sponsor, worked together to design, establish and develop a teleproctoring program to enable continuation of TLD bronchoscopy procedures in the UK despite travel restrictions associated with the covid-19 pandemic. The program involved a proctor teleproctoring the case from outside of the bronchoscopy suite using 2-way audiovisual equipment that was set up to allow visualization of the bronchoscopic view of the patients’ airways, the bronchoscopist themselves including their hand positions, and where relevant the fluoroscopy screen.

A retrospective analysis of anesthesia, bronchoscopy and fluoroscopy duration recorded at the time of the procedures was performed comparing teleproctoring procedures to those performed face-to-face. The safety of the procedure was evaluated looking at complication rates and rates of device deficiencies.

### Quantification and statistical analysis

Baseline demographics and clinical characteristics are presented as averages with standard deviations. Baseline characteristics between the groups were compared using Chi-squared test for categorical variables, unpaired T-test for parametric continuous data and Mann-Whitney test for non-parametric continuous data. Comparisons in outcomes between the two groups were made using unpaired T-tests for continuous parametric data and Mann-Whitney test for continuous non-parametric data and Fisher’s exact test for categorical data. Statistical analysis was performed at the 0 · 05 significance level with 95% confidence intervals using RStudio (version 2022.07.2) and R (version 4.2.2).

### Additional resources

The AIRFLOW-3 trial is registered on ClinicalTrials.gov (NCT03639051).
